# Pre-Treatment Neutrophil-to-Lymphocyte Ratio (NLR) as a Predictive Marker of Pazopanib Treatment for Soft-Tissue Sarcoma

**DOI:** 10.3390/cancers13246266

**Published:** 2021-12-14

**Authors:** Yasuyoshi Sato, Kenji Nakano, Xiaofei Wang, Naoki Fukuda, Tetsuya Urasaki, Akihiro Ohmoto, Naomi Hayashi, Mayu Yunokawa, Makiko Ono, Junichi Tomomatsu, Masanori Saito, Yusuke Minami, Keiko Hayakawa, Yuki Funauchi, Taisuke Tanizawa, Keisuke Ae, Seiichi Matsumoto, Shunji Takahashi

**Affiliations:** 1 Department of Medical Oncology, The Cancer Institute Hospital of Japanese Foundation for Cancer Research, Tokyo 135-8550, Japan; yasuyoshi_s@hotmail.com (Y.S.); gyohi.oh@jfcr.or.jp (X.W.); naoki.fukuda@jfcr.or.jp (N.F.); tetsuya.urasaki@jfcr.or.jp (T.U.); akihiro.omoto@jfcr.or.jp (A.O.); naomi.hayashi@jfcr.or.jp (N.H.); mayu.yunokawa@jfcr.or.jp (M.Y.); makiko.ono@jfcr.or.jp (M.O.); junichi.tomomatsu@jfcr.or.jp (J.T.); s.takahashi-chemotherapy@jfcr.or.jp (S.T.); 2 Department of Orthopedic Oncology, The Cancer Institute Hospital of Japanese Foundation for Cancer Research, Tokyo 135-8550, Japan; masanori.saito@jfcr.or.jp (M.S.); yusuke.minami@jfcr.or.jp (Y.M.); hayakawakeiko@mac.com (K.H.); yuki.funauchi@jfcr.or.jp (Y.F.); taisuke.tanizawa@jfcr.or.jp (T.T.); keisuke.ae@jfcr.or.jp (K.A.); smatsumoto@jfcr.or.jp (S.M.)

**Keywords:** neutrophil-to-lymphocyte ratio, NLR, pazopanib, soft-tissue sarcoma, STS

## Abstract

**Simple Summary:**

Second-line systematic therapy options for soft-tissue sarcoma (STS) have remained unchanged for decades due to the rarity and various histological types associated with STS. Challenges with molecular-targeted treatments for STS led to the approval of pazopanib and its wide use for STS. However, predictive markers of pazopanib treatment for STS have not been identified. Baseline neutrophil-to-lymphocyte ratio (NLR) is known as a candidate biomarker for several cancers. In this retrospective study, we investigated the use of the NLR as a predictor for the efficacy and prognosis of pazopanib in patients with STS. Our findings could be useful for the development of biomarker-targeted therapies for STS.

**Abstract:**

Pazopanib with trabectedin and eribulin is widely used to treat soft-tissue sarcoma (STS). We have shown that baseline neutrophil-to-lymphocyte ratio (NLR) may predict the efficacy and patient prognosis of eribulin. Changes in NLR, but not baseline NLR, can predict patient prognosis of trabectedin. However, prognostic factors of pazopanib for STS have not been identified. We present a retrospective analysis of 141 patients treated with pazopanib for recurrent or metastatic non-round cell STS. Univariate and multivariate analyses were performed to determine the predictive factors of durable clinical benefit (DCB), overall survival (OS), and progression-free survival. L-sarcoma histology (odds ratio [OR] = 0.31, 95% CI = 0.12–0.79; *p* = 0.014) and pre-treatment NLR < 3.0 (OR = 2.03, 95% CI = 1.02–6.67; *p* = 0.045) were independent predictive factors of DCB. Pre-treatment NLR < 3.0 (hazard ratio [HR] = 0.55, 95% CI = 0.36–0.84; *p* = 0.0057), liposarcoma histology (HR = 1.78, 95% CI = 1.09–2.91; *p* = 0.022), primary extremity site (HR = 0.48, 95% CI = 0.31–0.75; *p* = 0.0010), ECOG PS ≥ 1 (HR = 1.62, 95% CI = 1.08–2.42; *p* = 0.019), and CRP < 0.3 (HR = 0.52, 95% CI = 0.33–0.82; *p* = 0.0050) were independent predictive factors of OS. These findings indicate that baseline NLR predicts the efficacy and patient prognosis of pazopanib for STS.

## 1. Introduction

Pazopanib is an oral multitargeted tyrosine kinase inhibitor (TKI) that targets vascular endothelial growth factor (VEGF) receptors (VEGFR)−1, −2, and −3, platelet-derived growth factor receptors (PDGFR)-α and -β, and c-kit [1].

A randomized, double-blind phase III trial (VEG105192) in treatment-naive and cytokine-pre-treated patients with advanced renal cell carcinoma (RCC) showed significant improvement of progression-free survival (PFS) and tumor response for pazopanib compared with placebo [2]. From these results, pazopanib was first approved for the treatment of advanced RCC in the United States in October 2009 and in Europe in June 2010. 

A randomized, double-blind phase III trial (PALETTE) was performed in patients with metastatic soft-tissue sarcoma (STS), excluding patients with any type of adipocytic sarcoma and gastrointestinal stromal tumors, after failure of standard chemotherapy [3]. The findings demonstrated that pazopanib had a longer median PFS of 4.6 months compared with 1.6 months for placebos (*p* < 0.0001) [3]. These results supported the approval of pazopanib for clinical use in the United States in April 2012 and in Europe in August 2012. Additionally, analysis of the Japanese subpopulation in the PALETTE trial showed a median PFS of 24.7 weeks for patients treated with pazopanib, compared with a PFS of 7.0 weeks for patients receiving the placebo (*p* = 0.002) [4]. From these results, pazopanib was approved for the treatment of all types of STS in Japan in September 2012. Despite these advances, predictive markers for pazopanib in patients with STS have not been identified. In addition to pazopanib, eribulin and trabectedin are also used as second- or later-line treatment options for STS. 

The neutrophil-to-lymphocyte ratio (NLR), which is defined as the absolute neutrophil count (ANC) divided by the absolute lymphocyte count (ALC) of peripheral blood, is a marker of systemic inflammation, and a higher NLR is indicative of poor prognosis in several cancers [5,6,7]. Our recent retrospective study reported that in patients with STS, a low pre-treatment NLR could act as a predictive marker of PFS and durable clinical benefit (DCB) for patients receiving eribulin [8]. Additionally, changes in the NLR, but not baseline NLR, could act as an independent predictor for OS in patients treated with trabectedin [9]. However, little is known about the association between NLR and pazopanib monotherapy for STS. Herein, we explore factors that predict the efficacy of pazopanib, including NLR, for patients with STS and specifically non-round cell sarcoma.

## 2. Patients and Methods

### 2.1. Patients 

We retrospectively analyzed prospectively collected data from 141 patients with recurrent or metastatic non-round cell STS who began treatment with pazopanib at the Cancer Institute Hospital of the Japanese Foundation for Cancer Research (JFCR) between December 2012 and December 2019. The database comprised the following patient characteristics: age, sex, histological diagnosis, primary tumor location, ECOG PS, number of previous systemic chemotherapies, and the ANC, ALC, and CRP of blood samples collected before the first infusion. These factors were categorized as follows: age: <40 years, ≥40 years, or <65 years, ≥65 years; histology: L-sarcoma (leiomyosarcoma and liposarcoma) or non-L-sarcoma or Liposarcoma or non-liposarcoma; primary tumor location: extremities or non-extremities; ECOG PS: 0 or ≥1; number of previous systemic chemotherapies: 0–1 or ≥2; ALC: <1500/μL or ≥1500/μL; NLR (calculated as ANC divided by ALC): <3.0 or ≥3.0, and; CRP: <0.3 mg/dL or ≥0.3 mg/dL.

Pazopanib was initially administered at a daily dose of 800 mg in all patients. Dose reductions were permitted at the physician’s discretion. Dosing was adjusted or discontinued depending on the condition of each patient. All treatment was continued until the occurrence of unacceptable adverse effects or disease progression.

### 2.2. Statistical Analysis 

PFS and OS were estimated using the Kaplan–Meier method and the log-rank test. Data were censored on 31 August 2021. Patients who were lost to follow-up were censored at the date of last contact or follow-up. PFS was calculated from the date of pazopanib initiation to the date of disease progression or death from any cause. OS was calculated from the date of pazopanib initiation to the date of death from any cause. Patients who were alive on 31 August 2021 were censored for OS analysis. Tumor response was evaluated according to the Response Evaluation Criteria in Solid Tumors, version 1.1 [10], based on computed tomography (CT) findings. The best overall response was assessed as complete response (CR), partial response (PR), stable disease (SD), non-CR/non-PD, or progressive disease (PD). Patients with clinically progressed disease status were defined as PD without undergoing a CT scan in this study.

The overall response corresponded to the sum of the CR and PR, and disease control corresponded to the sum of the CR, PR, and SD rates. DCB was defined as CR, PR, SD, or non-CR/non-PD that lasted more than six months. We performed univariate and multivariate analyses to estimate potential prognostic factors for PFS, OS, and DCB; we calculated HRs using a Cox proportional hazards model for PFS and OS, and a logistic regression analysis for DCB. The two-sided level of significance was set to *p <* 0.1 for the univariate analysis and *p <* 0.05 for the multivariate analysis. Since ALC is used to calculate the NLR, when both values indicated a *p* < 0.1 in the univariate analysis, the value with the lower *p*-value was used in the multivariate analysis. Moreover, receiver operating characteristics (ROC) curves of ANC, ALC, and NLR at baseline in terms of prediction for longer OS than the median were performed, and the results are shown as area under curves (AUC). All statistical analyses were performed with EZR (Saitama Medical Center, Jichi Medical University, Saitama, Japan), which is a graphical user interface for R (The R Foundation for Statistical Computing, Vienna, Austria); specifically, it is a modified version of R commander designed to add statistical functions that are frequently used in biostatistics [11].

## 3. Results

### 3.1. Patient Characteristics 

A total of 141 patients with non-round cell STS were treated with pazopanib between December 2012 and December 2019. The 141-patient cohort included 73 men and 68 women, and the median age was 54 years (range = 19–85). The median duration of observation was 11.0 months (range = 0.8–293.0). In total, 132 patients had received doxorubicin as a perioperative or an earlier-line chemotherapy, regardless of histological subtype. Patient characteristics are shown in Table 1. Of the 141 patients, one (1%) had no available baseline blood cell count data. The median pre-treatment ANC was 3560/µL (range = 1030–44,420/µL), the ALC was 1070/µL (range = 80–2750/µL), and the median NLR was 3.50/µL (range = 0.88–31.15/µL).

### 3.2. Clinical Efficacy of Pazopanib 

**Figure 1:**
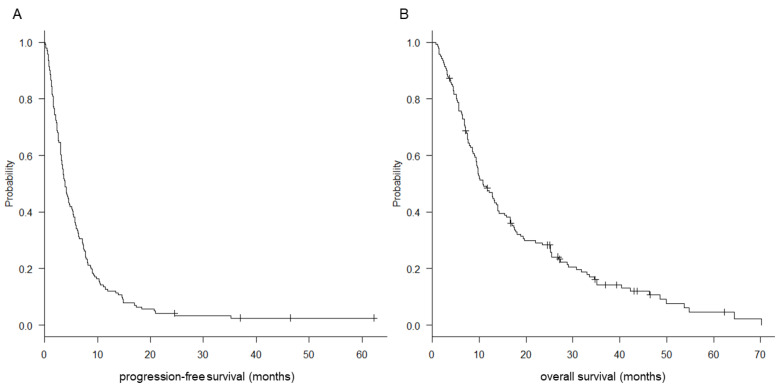
Kaplan–Meier curves for progression-free (**A**) and overall (**B**) survival of patients treated with pazopanib for non-round cell soft-tissue sarcoma (*n* = 141).

The objective response rate was 6% (*n* = 8) and the DCB rate was 32% (*n* = 45) in patients treated with pazopanib (Table 2). Pazopanib was withdrawn in one patient without evaluation of response due to a deterioration in their general condition. The median PFS and OS were 3.9 (95% confidence interval [CI] = 3.2–4.8) and 10.9 (95% CI = 9.3–13.9) months, respectively (Figure 1). 

### 3.3. Predictive Factors for DCB, PFS, and OS 

**Figure 2:**
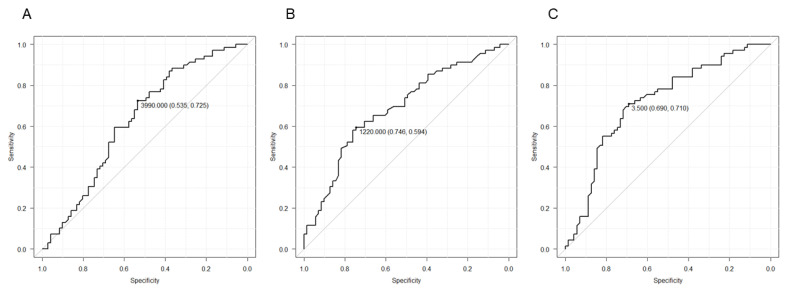
Receiver operating characteristic (ROC) curves for baseline absolute neutrophil count (**A**), absolute lymphocyte count (**B**), and neutrophil-to-Lymphocyte ratio (**C**).

As shown in Table 3, multivariate analysis indicated that L-sarcoma histology (odds ratio [OR] = 0.31, 95% CI = 0.12–0.79; *p* = 0.014) and pre-treatment NLR < 3.0 (OR = 2.03, 95% CI = 1.02–6.67; *p* = 0.045) were independent predictors of DCB. Age, sex, Eastern Cooperative Oncology Group performance status (ECOG PS), primary lesion, ALC, and C-reactive protein (CRP) were not associated with DCB. Moreover, as shown in Table 4, multivariate analysis indicated that pre-treatment NLR < 3.0 (hazard ratio [HR] = 0.55, 95% CI = 0.36–0.84; *p* = 0.0057), liposarcoma histology (HR = 1.78, 95% CI = 1.09–2.91; *p* = 0.022), primary extremity site (HR = 0.48, 95% CI = 0.31–0.75; *p* = 0.0010), ECOG PS ≥ 1 (HR = 1.62, 95% CI = 1.08–2.42; *p* = 0.019), and CRP < 0.3 (HR = 0.52, 95% CI = 0.33–0.82; *p* = 0.0050) were independent predictors of OS. However, only L-sarcoma histology (HR = 1.61, 95% CI = 1.10–2.37; *p* = 0.015) was associated with PFS (Table 5). ROC curves generated for ANC, ALC, and NLR in terms of prediction for longer OS than median showed that AUC values were 0.63 (95% CI = 0.54–0.72) for ANC, 0.69 (95% CI = 0.60–0.77) for ALC, and 0.72 (95% CI = 0.63–0.80) for NLR (Figure 2).

## 4. Discussion

To our knowledge, no other study has evaluated NLR in pazopanib-treated patients with STS. In this study, we investigated predictive factors of pazopanib monotherapy for patients with STS, including ALC and NLR. Notably, we identified low pre-treatment NLR (<3.0) and non-L-sarcoma histology as independent predictors of DCB. Low pre-treatment NLR (<3.0), primary extremity site, and better PS (PS = 0) were also established as independent predictors of prolonged OS. 

Some previous reports have suggested that the NLR may reflect the antitumor immunity status. Rosenberg et al. [12] reported that neutrophils could promote tumor progression, whereas lymphocytes are associated with the elimination of tumor cells. Moreover, the NLR was reported to reflect the balance of the immune system [6] and the cytokine profile; cytokines activates cluster of differentiation (CD) 4^+^ and CD8^+^ T-lymphocytes that regulate antitumor immunity [13]. 

For an antitumor immune response to effectively cause malignant cell death, a series of stepwise processes called the “cancer-immunity cycle” must be initiated and expanded. During this process, VEGF is thought to inhibit the infiltration step of T cells into tumors [14]. Therefore, anti-VEGF drugs can potentially promote T-cell infiltration into tumors, and thereby induce the cancer-immunity cycle via VEGF itself or VEGFR inhibition. Moreover, VEGF-A expression was reported to be significantly higher in the high-NLR group compared with the low-NLR group in colon cancer patients [13]. Therefore, it is biologically plausible that the NLR, reflecting host antitumor immune status and angiogenesis, could predict prognosis or efficacy of treatment with TKIs that target VEGFR, such as pazopanib.

In the present study, low pre-treatment NLR (<3.0) was identified as an independent predictive marker for DCB and better OS in patients with STS treated with pazopanib. For some multitargeted TKIs (that target VEGFR), an association between pre-treatment NLR and prognosis has been reported. A previous study of 109 patients with metastatic RCC treated with sunitinib, an orally administered TKI targeting VEGFR and PDGFR, suggested that low pre-treatment NLR was associated with better PFS and OS [15]. In previous studies of patients with thyroid cancer treated with Lenvatinib, an oral TKI that targets VEGFR and PDGFR, NLR was found to be a prognostic marker for differentiated thyroid cancer [16] and anaplastic thyroid cancer [17]. Our data were consistent with these results.

In this study, L-sarcoma histology was inversely associated with DCB and PFS. In the phase II trial that preceded the PALETTE trial, the liposarcoma cohort closed after the first stage as a result of insufficient efficacy [18]. In Japan, pazopanib is also approved for liposarcoma, and 25 patients with liposarcoma were included in this study. In the univariate analyses, liposarcoma had a lower OR or DCB rate and a higher HR for PFS, although the *p*-value was higher than 0.1. This was probably due to the small number of patients Therefore, liposarcoma histology was not adopted as a factor in multivariate analyses. However, in the L-sarcoma cohort (*n* = 54), the inverse effect for DCB and PFS in liposarcoma cases may have been statistically detected. In addition, histological types in which pazopanib was expected to be effective for, such as synovial sarcoma (*n* = 16) and alveolar soft part sarcoma (*n* = 6), were included in the non-L-sarcoma cohort, which could have contributed to the higher efficacy in the non-L-sarcoma cohort.

Several limitations of this study should be acknowledged. First, this was a retrospective study from a single institution, and selection bias may have resulted from physician or institution subjectivity when determining which patients should receive pazopanib at which line. Second, the NLR value is variable, not only by tumor type or immunity factors, but also by infection, corticosteroids, radiotherapy, or other physiological stresses. In this study, all patients did not have immunodeficiency, history of transplantation, and active infections. However, 64 out of 141 patients (45%) underwent prior radiotherapy. Although we used an NLR cut-off value of 3.0 in accordance with the findings, the appropriate cut-off value is still under debate. In summary, multicenter and prospective studies are warranted.

## 5. Conclusions

In conclusion, this retrospective study uncovered predictive factors of pazopanib monotherapy for STS patients. Notably, we found that low pre-treatment NLR (<3.0) and non-L-sarcoma histology were independent predictors of DCB, and that low baseline NLR (<3.0), primary extremity site, and better PS (PS = 0) were independent predictors of prolonged OS.

## Figures and Tables

**Table 1:** Baseline characteristics of the study patients (*n* = 141).

Characteristic	Category	*n* (%)
Age	≥40 years	105 (74)
	≥65 years	24 (17)
Gender	Male	73 (52)
Histology	L-sarcoma	54 (38)
	Liposarcoma	25 (18)
Location of primary lesion	Extremity	46 (33)
ECOG PS	0	80 (57)
	≥1	61 (43)
No. of previous chemotherapies	0	9 (6)
	1	54 (38)
	≥2	78 (55)
pre-ALC	≥1500 cells/μL	30 (21)
	<1500 cells/μL	110 (78)
	Unevaluated	1 (1)
pre-NLR	≥3.0	82 (58)
	<3.0	58 (41)
	Unevaluated	1 (1)
pre-CRP	≥0.3 mg/dL	74 (52)
	<0.3 mg/dL	66 (47)
	Unevaluated	1 (1)

ALC: absolute lymphocyte count; CRP: C-reactive protein ECOG PS: Eastern Cooperative Oncology Group performance status; NLR: neutrophil-to-lymphocyte ratio.

**Table 2:** Efficacy of pazopanib monotherapy in the study patients (*n* = 141).

		*n* (%)
Best overall response	CR	0 (0)
	PR	8 (6)
	SD	62 (44)
	Non-CR/non-PD	4 (3)
	PD	66 (47)
	Not evaluable	1 (1)
Objective response	CR + PR	8 (6)
Disease control	CR + PR + SD	70 (50)
Durable clinical benefit		45 (32)

CR: Complete response; PD: progressive disease; PR: partial response; SD: stable disease.

**Table 3:** Univariate and multivariate analyses of factors associated with durable clinical benefit.

Characteristic	Category	Univariate		Multivariate	
		Odds Ratio (95% CI)	*p*-Value	Odds Ratio (95% CI)	*p*-Value
Age	≥40 vs. <40 Years	1.09 (0.48–2.47)	0.84		
	≥65 vs. <65 Years	1.28 (0.60–2.74)	0.54	0.86 (0.35–2.12)	0.74
Gender	Male vs. female	0.74 (0.36–1.51)	0.41	0.87 (0.38–1.99)	0.74
Histology	L-Sarcoma vs. other	0.34 (0.15–0.76)	**0.0088**	0.31 (0.12–0.79)	**0.014**
	liposarcoma vs. other	0.48 (0.17–1.36)	0.17		
Primary lesion	Extremity vs. other	1.62 (0.77–3.40)	0.20		
ECOG PS	≥1 vs. 0	0.72 (0.35–1.48)	0.37		
No. of previous chemotherapies	≥2 vs. 0–1	0.41 (0.19–0.86)	**0.018**	0.47 (0.21–1.08)	0.075
ALC	<1500 vs. ≥1500/μl	1.41 (0.64–3.10)	0.39		
NLR	<3.0 vs. ≥3.0	2.35 (1.14–4.84)	**0.021**	2.61 (1.02–6.67)	**0.045**
CRP	<0.3 vs. ≥0.3 mg/dl	2.15 (1.05–4.44)	**0.038**	2.03 (0.83–4.97)	0.123

ALC: absolute lymphocyte count; CI, confidence interval; CRP: C-reactive protein; ECOG PS: Eastern Cooperative Oncology Group performance status; NLR: neutrophil-to-lymphocyte ratio. Statistically significant *p*-values are shown in bold.

**Table 4:** Univariate and multivariate analyses of factors associated with overall survival.

Characteristic	Category	Univariate		Multivariate	
		HR (95% CI)	*p*-Value	HR (95% CI)	*p*-Value
Age	≥40 vs. <40 Years	0.91 (0.61–1.37)	0.66		
	≥65 vs. <65 Years	0.80 (0.54–1.19)	0.27	0.84 (0.56–1.25)	0.397
Gender	Male vs. female	0.92 (0.65–1.32)	0.66	0.87 (0.59–1.29)	0.492
Histology	L-Sarcoma vs. other	1.05 (0.73–1.51)	0.799		
	liposarcoma vs. other	1.49 (0.93–2.38)	**0.096**	1.78 (1.09–2.91)	**0.022**
Primary lesion	Extremity vs. other	0.48 (0.32–0.72)	**<0.001**	0.48 (0.31–0.75)	**0.0010**
ECOG PS	≥1 vs. 0	1.52 (1.06–2.18)	**0.024**	1.62 (1.08–2.42)	**0.019**
No. of previous chemotherapies	≥2 vs. 0–1	1.20 (0.84–1.73)	0.32		
ALC	<1500 vs. ≥1500/μL	0.61 (0.41–0.92)	0.020		
NLR	<3.0 vs. ≥3.0	0.45 (0.30–0.65)	**<0.001**	0.55 (0.36–0.84)	**0.0057**
CRP	<0.3 vs. ≥0.3 mg/dL	0.37 (0.25–0.55)	**<0.001**	0.52 (0.33–0.82)	**0.0050**

ALC: absolute lymphocyte count; CI, confidence interval; CRP: C-reactive protein; ECOG PS: Eastern Cooperative Oncology Group performance status; HR, hazard ratio; NLR: neutrophil-to-lymphocyte ratio. Statistically significant *p*-values are shown in bold.

**Table 5:** Univariate and multivariate analyses of factors associated with progression-free survival.

Characteristic	Category	Univariate		Multivariate	
		HR (95% CI)	*p*-Value	HR (95% CI)	*p*-Value
Age	≥40 vs. <40 Years	0.93 (0.63–1.36)	0.706		
	≥65 vs. <65 Years	0.73 (0.51–1.06)	0.099	0.70 (0.48–1.02)	0.062
Gender	Male vs. female	1.21 (0.86–1.70)	0.272	1.18 (0.82–1.69)	0.38
Histology	L-Sarcoma vs. other	1.56 (1.10–2.23)	**0.014**	1.61 (1.1–2.37)	**0.015**
	liposarcoma vs. other	1.70 (1.09–2.65)	**0.02** **0**		
Primary lesion	Extremity vs. other	0.69 (0.47–0.99)	**0.043**	0.76 (0.51–1.13)	0.179
ECOG PS	≥1 vs. 0	1.36 (0.97–1.92)	**0.074**	1.39 (0.96–2.01)	0.086
No. of previous chemotherpies	≥2 vs. 0–1	1.23 (0.87–1.73)	0.236		
ALC	<1500 vs. ≥1500/μL	0.86 (0.59–1.26)	0.438		
NLR	<3.0 vs. ≥3.0	0.74 (0.52–1.04)	**0.082**	0.78(0.53–1.16)	0.219
CRP		0.62 (0.44–0.87)	**0.006**	0.7(0.47–1.05)	0.083

ALC: absolute lymphocyte count; CI, confidence interval; CRP: C-reactive protein; ECOG PS: Eastern Cooperative Oncology Group performance status; HR, hazard ratio; NLR: neutrophil-to-lymphocyte ratio. Statistically significant *p*-values are shown in bold.

## Data Availability

The data presented in this study are available on request from the corresponding author. The data are not publicly available due to legal constraints.
